# Case Report: Post-gastrectomy reactive hyperinsulinemic hypoglicaemia: glucose trends before and after canagliflozin treatment

**DOI:** 10.3389/fendo.2023.1193696

**Published:** 2023-08-14

**Authors:** G. Bellastella, P. Caruso, C. Carbone, M. di Nuzzo, L. Scappaticcio, V. Amoresano Paglionico, M. I. Maiorino, K. Esposito

**Affiliations:** ^1^ Unit of Endocrinology and Metabolic Diseases, University of Campania “Luigi Vanvitelli”, Naples, Italy; ^2^ Department of Advanced Medical and Surgical Sciences, University of Campania Luigi Vanvitelli, Naples, Italy

**Keywords:** canagliflozin, SGLT2, SGLT1, hypoglycaemia, FGM (flash glucose monitoring), obesity

## Abstract

The pathogenesis of post-gastrectomy reactive hyperinsulinaemic hypoglycaemia is not yet fully clarified. Recent studies suggest an up-regulation of the intestinal glucose transporter SGLT-1 aimed to prevent carbohydrate malabsorption. The overexpression of SGLT-1 could therefore represents one of the mechanisms underlying the wide glycemic excursions found in patients after gastrectomy, but studies investigating the use of SGLT-1/SGLT-2 inhibitors in patients with post-gastrectomy reactive hyperinsulinemic hypoglycaemia are very scant in the literature. We report the case of a 37-year-old non diabetic man who frequently presented symptoms of hypoglycaemia in the postprandial period. In 2012, he underwent Roux en-Y gastric bypass (RYGB) and after two years, he started to experience typical symptoms of reactive hyperinsulinaemic hypoglycaemia. We suggested healthy modifications of dietary habits and periodic follow-up visits with a dietitian. After three months, the patient still presented symptoms of reactive hypoglycaemia; we provided him with Flash Glucose Monitoring (FGM) to assess trend of glucose levels in interstitial fluid during the day and we decided to introduce canagliflozin 300 mg/day before the main meal. Hypoglycaemic events previously referred by the patient and clearly recorded by FGM completely disappeared taking canagliflozin. We found a reduction of time spent in hypoglycaemia, an improvement of glycemic variability and an increase of time in target range. It was also noted a reduction of time spent in hyperglicemia with consequent improvement of average glucose values and of glucose main indicator. This is the first report with FGM supporting a role of canagliflozin in the management of post-gastrectomy reactive hyperinsulinaemic hypoglycaemia. Our preliminary results are very limited but in line with those of the literature and showed for the first time a reduction of hypoglycaemic events and an improvement of glycemic variability through a flash glucose monitoring system. Further studies are mandatory to confirm this therapeutic opportunity.

## Background

The pathogenesis of post-gastrectomy reactive hyperinsulinaemic hypoglycaemia is not yet fully clarified, but different mechanisms are involved and the increased incretin response due to a rapid and marked glycemic excursion is already well documented (1). Recent studies suggest an adaption of absorptive function of intestinal mucosa in response to the anatomical modification of the gastrointestinal tract with up-regulation of the intestinal glucose transporter SGLT-1 (Sodium Glucose Cotransporter 1) aimed to prevent carbohydrate malabsorption (2, 3).

The overexpression of SGLT-1 could therefore represents one of the mechanisms underlying the wide glycemic excursions found in patients after gastrectomy surgery. Despite this, the studies that investigated the use of SGLT-1/SGLT-2 inhibitors (like canagliflozin) in patients with post-gastrectomy reactive hyperinsulinaemic hypoglycaemia are very scant in the literature.

## Case description

We report the case of a 37-year-old non diabetic man who frequently presented symptoms of hypoglycaemia in the postprandial period. In 2012, he underwent bariatric surgery, specifically Roux-en-Y gastric bypass (RYGB), that led to a body weight loss of 60 kilograms.After two years, the patient started to experience symptoms like sweating, tachycardia, confusion and generalized weakness after meals, particularly if these were rich in carbohydrates. This was the classic presentation of reactive hyperinsulinaemic hypoglycaemia, since the patient showed a blood glucose value of 26 mg/dl 120 minutes after a 75-g oral glucose tolerance test (OGTT) with an insulin peak greater than 500 µU/ml at 60 minutes. His glycated hemoglobin A1c was 5.4% and other causes of hypoglycaemia were excluded. A careful history and a full laboratory assessment were performed. In particular we excluded alcohol intake and iatrogenic or autoimmune hypoglycaemia through insulin, c-peptides, insulin autoantibodies and urinary sulfanilureas concentrantions assay. Hormonal deficiencies were excluded since GH,IGF-1, and cortisol concentrations were in the normal range (GH 6,67 ng/ml, IGF-1 192,1, cortisol 14,3 ng/ml). We found no pathological values suggestive for acute or chronic illness on blood tests, neither signs on physical examination. Furthermore the patient showed normal liver and kidney functions.

As a first approach we suggested healthy modifications of dietary habits and recurrent follow-up visits with a dietitian. We prescribed a diet based on small and frequent meals, with complex carbohydrates mixed to fibers, proteins and healthy fats, suggesting to avoid simple sugars with high-glycemic index. After three months, the patient still presented with symptoms of reactive hypoglycaemia; we provided him with Flash Glucose Monitoring (FGM, Freestyle libre 2) to assess trend of glucose levels in interstitial fluid during the day. We decided to prescribe oral canagliflozin therapy (at a dose of 300 mg/day; 1 tablet taken before the richest carbohydrate meal of the day, i.e. lunch). Therefore, we compared three consecutive days with nutritional approach alone with three consecutive days of treatment with canagliflozin and recorded and analyzed the FGM data of two study periods. Since canagliflozin could also affect body weight and blood pressure through a loss of calories and osmotic diuresis with urinary glucose excretion, after 14 days of treatment with canagliflozin we evaluated vital signs and the quality of life by a modified version of the hypoglycaemia Fear Survey-II questionnaire. The possible occurrence of adverse events related to the use of canagliflozin was also investigated. The patient was properly informed about the off-label use of canagliflozin for management of post-gastrectomy reactive hyperinsulinaemic hypoglicaemia and a signed, informed consent was obtained before prescribing canagliflozin.

**Figure 1:**
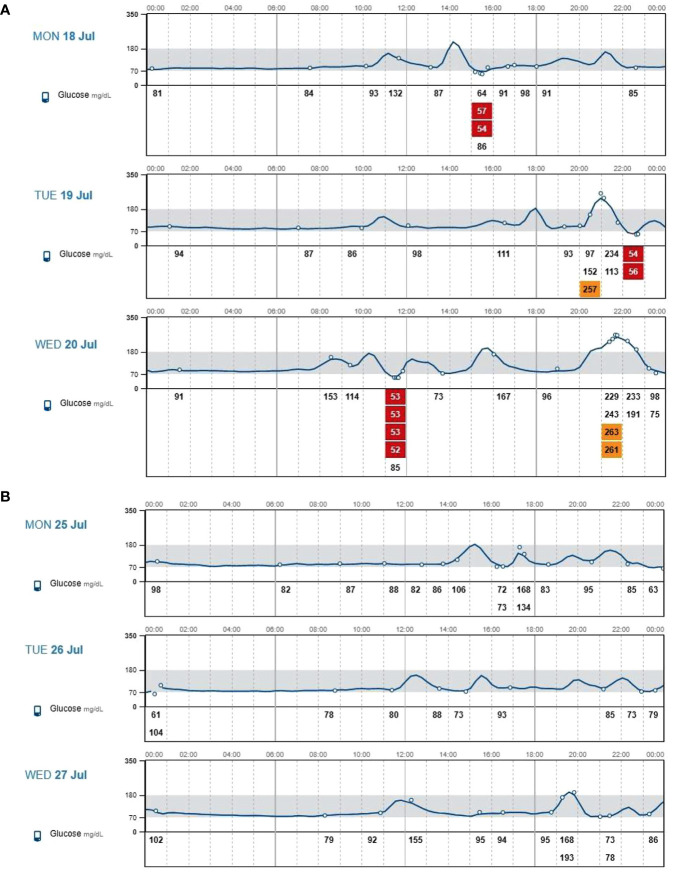
**(A)** Interstitial glucose values assessed through flash glucose monitoring during three consecutive days with nutritional approach alone. **(B)** Interstitial glucose values assessed through flash glucose monitoring during three consecutive days with nutritional approach plus 300-mg canagliflozin treatment.

The main FGM metrics referring to the three consecutive days with nutritional approach alone and to the three consecutive days following the addition of canagliflozin are reported in **Figures 1A, B**. Severe hypoglycemic episodes (time below range <54 mg/dL) disappeared, whereas there was a substantial reduction in less severe hypoglycemic episodes. Furthermore we found a reduction of time spent in hypoglycaemia (time below range <54 mg/dL, from 2.9% to 0.0%; time below range 54-70 mg/dL, from 8.8% to 3.1%), a reduction of glycemic variability (expressed by the coefficient of variation, from 48.1% to 24.1%) and an increase of time spent in range 70-180 mg/dL (from 76.5% to 96.9%) (**Tables 1**, **2**). Contextually there was a reduction of time spent in hyperglycemia (time above range 180-250 mg/dl, from 5.9% to 0%; time above range >250 mg/dl, from 5,9% to 0%) with subsequent improvement of average glucose values (from 111,7 mg/dl to 92,4 mg/dl) and of glucose main indicator (GMI, from 6,0% to 5,5%). During a 3-months follow up period our patient presented steady values of blood pressure and he reported a further weight loss of three kilograms, also following a balanced diet. Moreover, he did not experience urinary infections, typical side effect of the drug. The patient reported a significant improvement in quality of life after 14 days of canagliflozin treatment as evaluated by a modified version of the hypoglycaemia Fear Survey-II questionnaire.

**Table 1:** Main FGM metrics during three consecutive days with nutritional approach alone.

**Time above range,%** **>250 mg/dL**	5,9
**Time above range,%** **180-50 mg/dL**	5,9
**Time in range, %** **70-180 mg/dL**	76,5
**Time below range, %** **54-70 mg/dL**	8,8
**Time below range, %** **< 54 mg/dL**	2,9
**Time sensor active,%**	100,0
**Avarage glucose, mg/dL**	111,7
**SD**	53,7
**CV, %**	48,1
**GMI, %**	6,0

CV coefficient of variability; GMI,Glucose managment indicator; SD, standard deviation.Time In Range 70-180 mg/dl = green. Time Above Range 180-250 mg/dl = yellow. Time Above Range > 250 mg/dl = orange. Time Below Range 54-70 mg/dl= rose.Time Below Range < 54 mg/dl = red.

**Table 2:** Main FGM metrics during three consecutive days with nutritional approach plus 300-mg canagliflozin treatment.

**Time above range,%** **>250 mg/dL**	0,0
**Time above range,%** **180-50 mg/dL**	0,0
**Time in range, %** **70-180 mg/dL**	96,9
**Time below range, %** **54-70 mg/dL**	3,1
**Time below range, %** **< 54 mg/dL**	0,0
**Time sensor active,%**	100,0
**Avarage glucose, mg/dL**	92,4
**SD**	22,3
**CV, %**	24,1
**GMI, %**	5,5

CV, coefficient of variability; GMI, Glucose managment indicator; SD, standard deviation.Time In Range 70-180 mg/dl = green. Time Above Range 180-250 mg/dl = yellow. Time Above Range > 250 mg/dl = orange. Time Below Range 54-70 mg/dl= rose.Time Below Range < 54 mg/dl = red.

## Discussion

This is the first report with FGM supporting a role of canagliflozin in the management of post-gastrectomy reactive hyperinsulinaemic hypoglycaemia.

Canagliflozin is one of the SGLT-2 inhibitors approved for the treatment of type 2 diabetes mellitus. It is also able to exert a modest inhibition of SGLT-1 expressed on the luminal side of the enterocytes at higher clinical dosages (>200 mg) (4). Accordingly, canagliflozin could reduce postprandial glucose and insulin spikes by delaying intestinal glucose absorption (5, 6). Currently, few studies have been conducted in subjects undergoing gastrointenstinal tract surgery, in particular gastric bypass according to the Roux-en-Y technique. In a randomized, controlled, crossover study, 10 participants who underwent Roux-en-Y-gastric bypass (RYGB) were investigated on 2 days separated by at least 7 days with ingestion of a glucose drink containing 50 g of glucose dissolved in 200 mL of water, with or without acute pretreatment with 600 mg of canagliflozin. The results of the study showed how the administration of canagliflozin (at a dose of 600 mg) determined a reduction in postprandial blood glucose, insulin and glucagon-like peptide 1 (GLP-1) peaks suggesting that intestinal glucose absorption through SGLT-1 contributes to determine the increased incretin response occurring after glucose ingestion (7).

Subsequently, Ciudin et al. (8) studied the effect of administration of 300 mg canagliflozin by evaluating 21 patients undergoing RYGB with a 100-g oral glucose tolerance test performed at baseline and after 2 weeks of canagliflozin treatment; OGTT after canagliflozin treatment showed a significant reduction of plasma glucose and insulin levels. At 180 minutes, a significant reduction (85.7%) of the rate of hypoglycaemia was observed after canagliflozin treatment (p < 0.00001). The authors noted that Canagliflozin (300 mg) significantly decreased glucose absorption and prevented hyperinsulinaemic hypoglycaemia after 100 g OGTT in patients with RYGB. Our preliminary results are very limited but in line with those of the literature and showed for the first time a reduction of hypoglycemic events and an improvement of glycemic variability through a flash glucose monitoring system in a patient with postgastrectomy reactive hyperinsulinaemic hypoglycaemia treated with canagliflozin. Other effects of canagliflozin may contribute to these results (7); indeed it determines glycosuria which could help to reduce post-prandial hypoglycemic episodes by itself and by stimulating the secretion of glucagon. Further studies are mandatory to confirm and clarify this therapeutic opportunity.

## Data availability statement

The raw data supporting the conclusions of this article will be made available by the authors, without undue reservation.

## Ethics statement

Ethical approval was not provided for this study on human participants because this is a single case report. The patients/participants provided written informed consent to participate in this study. Written informed consent was obtained from the participant/patient(s) for the publication of this case report.

## Author contributions

Study conception and design: KE, GB; data collection: PC; analysis and interpretation of results: MIM, LS; draft manuscript preparation: CC, MdN, VAP. All authors reviewed the results and approved the final version of the manuscript.
